# Predictors of Body Temperature in Nose-Horned Viper (*Vipera ammodytes*) Across Different Populations

**DOI:** 10.3390/ani16081239

**Published:** 2026-04-17

**Authors:** Mladen Zadravec, Roman Cesarec, Bartol Smutni, Mario Zadravec, Tomislav Gojak, Marko Glogoški, Duje Lisičić

**Affiliations:** 1 Ministry of Environmental Protection and Green Transition, Institute for Environment and Nature, Radnička cesta 80, HR-10000 Zagreb, Croatia; mladen.zadravec@mzozt.hr; 2 Department of State, U.S. Embassy Zagreb, Thomasa Jeffersona 2, HR-10000 Zagreb, Croatia; roman@snakebite.com; 3 Faculty of Agriculture, University of Zagreb, Svetošimunska 25, HR-10000 Zagreb, Croatia; bsmutni@gmail.com; 4 Independent Researcher, HR-10000 Zagreb, Croatia; mario.zadravec57@gmail.com; 5 Association Hyla, Lipovac I No. 7, HR-10000 Zagreb, Croatia; tomislav.gojak@hhdhyla.hr; 6 Department of Animal Physiology, Division of Biology, Faculty of Science, University of Zagreb, HR-10000 Zagreb, Croatia; marko.glogoski@biol.pmf.hr

**Keywords:** conservation, ectothermy, flexible thermal strategies, local adaptation, species-environment interactions, thermal biology

## Abstract

Body temperature regulation in ectotherms is influenced by numerous environmental, morphological, and physiological factors, some of which operate in population-specific ways. Understanding how these factors shape thermal biology is important for species conservation. The nose-horned viper, an ecologically significant yet understudied mesopredator of southeastern Europe and Asia Minor, occupies diverse ecosystems facing ongoing degradation. Over five years, we investigated how 12 environmental, behavioral, morphological, and physiological variables influenced field body temperature across three climatically distinct populations of nose-horned vipers. Using an information-theoretic approach with model averaging, we identified important predictors and assessed population-specific effects. Air temperature at 5 cm above the snake’s position, humidity, and wind were highly important predictors across all populations, whereas physiological states (shedding and digestion) exerted weaker effects. Microhabitat type and time of day emerged as highly important population-specific predictors, while body size showed weaker, population-dependent effects. Neither sex, cloud cover, nor behavioral state contributed meaningfully to model fit. Mean body temperatures were similar across populations and sexes. By integrating environmental, behavioral, physiological, and morphological variables, this study comprehensively identifies predictors of body temperature in nose-horned vipers. Site-tailored maintenance of structurally diverse habitats is essential for preserving thermoregulatory opportunities and ensuring long-term persistence of nose-horned vipers.

## Simple Summary

Animals that cannot produce their own body heat must carefully regulate body temperature to survive. Snakes face particular constraints because their elongate, limbless bodies specifically influence rates of heat exchange with the environment. We investigated which environmental and biological factors influence body temperature in nose-horned vipers across three populations inhabiting distinct habitats. We investigated field body temperatures alongside 12 environmental, morphological, behavioral, and physiological variables. We found that air temperature at 5 cm above the snake’s position, humidity, and wind affected body temperatures in all populations. In contrast, microhabitat use and the time when each individual was found exerted population-specific effects on body temperature. Despite these differences, mean body temperatures were similar across populations and sexes. This demonstrates that vipers achieve similar thermal outcomes through population-specific regulatory pathways. These findings underscore the importance of maintaining structurally diverse habitats that enable behavioral thermoregulation under changing environmental conditions. Preserving such habitats is essential for the species’ long-term persistence.

## 1. Introduction

Thermoregulation, the capacity of an organism to influence its body temperature, is fundamental to survival and physiological performance in many organisms [1,2,3]. In ectotherms, thermoregulation is governed by interacting internal (physiological, morphological) and external (environmental) factors. Because ectotherms cannot generate sufficient metabolic heat to maintain body temperature independently, they rely primarily on external heat sources [3,4]. Therefore, alterations in the thermal environment present substantial physiological and ecological challenges for ectotherms [5]. This is particularly true for terrestrial ectotherms in temperate zones, where pronounced diel and seasonal temperature fluctuations exist. These fluctuations pose a challenge for individuals to maintain body temperatures (T_b_) within ranges that support survival and optimal performance [3,6].

Among ectotherms, reptiles are particularly well-studied models for thermoregulatory research [7,8,9,10,11]. Recent studies have increasingly addressed how climate change affects reptilian thermoregulation, emphasizing species-specific adaptability and vulnerability to thermal stress [12,13,14]. Reptilian thermoregulation can be influenced by numerous interacting factors, including morphometry, physiology, behavior, and environmental conditions, which essentially act as predictors of T_b_. For instance, thermoregulatory patterns may differ between juveniles and adults, shift with feeding status, are affected by gravidity, and involve behavioral modifications mediated by habitat quality [7,8,15,16,17]. Indeed, behavioral thermoregulation is the primary mechanism by which reptiles regulate T_b_. For example, for warming up, a reptile usually moves to a suitable basking spot, whereas for cooling off, it retreats into the shade. Additionally, reptiles modulate solar heat gain by adjusting body orientation relative to the sun [8]. Behavioral thermoregulation is now recognized not as a set of isolated actions but as an integrated response reflecting thermal tolerances and physiological constraints modulated at the individual level. Despite complex, context-dependent variation in individual measurements, mean T_b_ often remains consistent across populations within a species [18,19,20]. Identifying T_b_ predictors thus provides a framework for interpreting population-level thermal variation and assessing vulnerability to climate warming.

The elongate, limbless body plan of snakes poses distinct thermoregulatory challenges, including high surface-area-to-volume ratios and direct contact with substrate, both accelerating heat exchange [21]. Moreover, snakes have historically received less research attention than lizards in thermoregulation studies [21]. Comparative studies indicate that behavioral thermal responses vary little among viper species, suggesting that conserved thermal tolerances constrain realized niches and geographic distributions [22,23]. At the same time, studies of South American and African vipers show that local conditions—particularly ambient temperature and habitat structure—strongly modulate behavioral responses, underscoring the importance of context-dependent adjustment over fixed strategies [23,24].

The nose-horned viper, *Vipera ammodytes* (Linnaeus, 1758), represents an excellent model species for further examination of these dynamics. Its broad climatic and habitat distribution, combined with strong reliance on microhabitat use, makes it well-suited for testing how field T_b_ is influenced by various internal and external predictors. *Vipera ammodytes* (Viperidae) is a venomous snake distributed from northeastern Italy and southern Austria across the Balkans to the Caucasus [25,26,27,28]. As a mesopredator, *V. ammodytes* plays a crucial role in ecosystem function, notably by regulating populations of small mammals, its primary prey across much of its range (e.g., [27,29,30,31]). Its broad distribution and ecological adaptability make understanding its thermoregulatory strategies particularly important for predicting thermoregulatory responses to environmental change and for informing effective conservation planning [8,32].

Research on environmental determinants of T_b_ in *V. ammodytes* remains limited, with two recent papers focusing only on the northeastern (NE) clade [20,33]. Čubrić and Crnobrnja-Isailović [33] found a significant correlation between T_b_ and environmental temperatures, but detected no differences among locations or between sexes. Dyugmedzhiev et al. [20] showed that *V. ammodytes* in the northeastern Balkans achieves thermoregulation through behavioral adjustments in microhabitat use and activity timing, yet no taxonomic, physiological, age-related, or sex-specific differences in field T_b_ were detected. Indeed, populations of the same species do not always respond uniformly to environmental conditions [13,34,35,36], and similar thermal outcomes can arise from different predictor combinations. How field T_b_ is shaped across geographically distinct populations of other *V. ammodytes* clades remains poorly understood.

This study examined how external (environmental) and internal (morphological, behavioral, and physiological) factors influence field T_b_ of both active and inactive snakes in three populations of this species’ NW clade inhabiting contrasting climatic and vegetation zones. We hypothesized that abiotic, morphological, sex-specific, and physiological characteristics would act as consistent T_b_ predictors across populations, whereas behavioral mechanisms (including microhabitat and time of day) would function as population-specific buffers compensating for local environmental differences. Consequently, we predicted that these distinct regulatory pathways would ultimately converge, resulting in a conserved mean T_b_ across populations and sexes.

## 2. Materials and Methods

### 2.1. Focal Species

**Figure 1:**
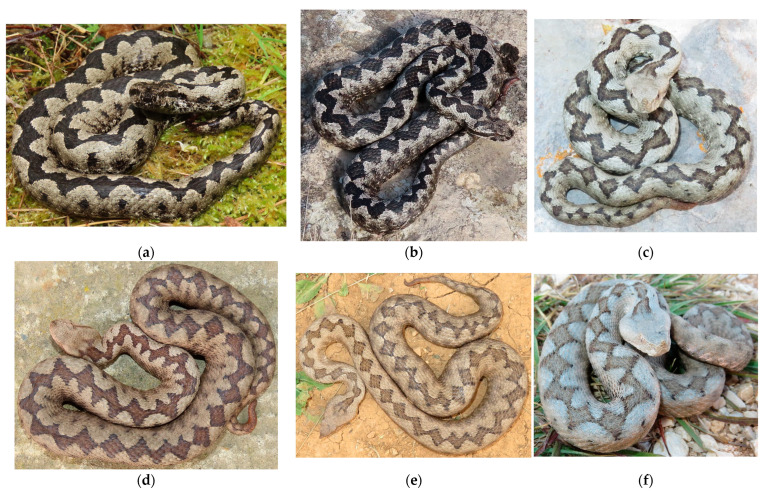
Photographs showing characteristic nose-horned viper (*Vipera ammodytes*) individuals from the three biogeographical regions of Croatia: (**a**) male from Bizek (Medvednica Mt.), (**b**) male from Lika (Malo Kamensko), (**c**) male from Vir Island, (**d**) female from Bizek (Medvednica Mt.), (**e**) female from Lika (Malo Kamensko), (**f**) female from Vir Island. All photos by Mladen Zadravec.

*Vipera ammodytes* is among the largest European vipers (Figure 1). Mean total length is approximately 65 cm; females average larger body sizes, though males attain greater maximum lengths [28,31]. Its elevational range spans from sea level to over 2000 m. It occupies diverse habitats, predominantly warm, dry areas such as rocky slopes, stone walls, and vegetated outcrops, but also cooler, more humid sites including open forests and montane grasslands [25,26,27,28]. The species is primarily diurnal but exhibits crepuscular and nocturnal activity when conditions allow [28,37]. Activity declines during midday, particularly in summer [31]. Females prefer slightly higher humidity than males [31]. The species avoids windy conditions (particularly wind speeds > 2 m/s) and cloud cover exceeding 60% [31]. Subspecific taxonomy has varied, with three to seven subspecies recognized in southeastern Europe [38,39]; the nominate subspecies, belonging to the northwestern (NW) clade, occurs in Croatia [19,40,41].

### 2.2. Study Sites

**Figure 2:**
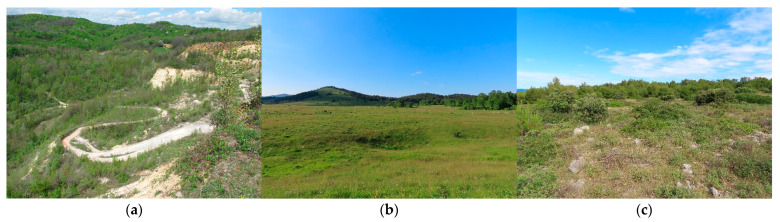
Photographs showcasing the three study sites: (**a**) Bizek (Medvednica Mt.), (**b**) Lika (Malo Kamensko), and (**c**) Vir Island. All photos by Mladen Zadravec.

**Figure 3:**
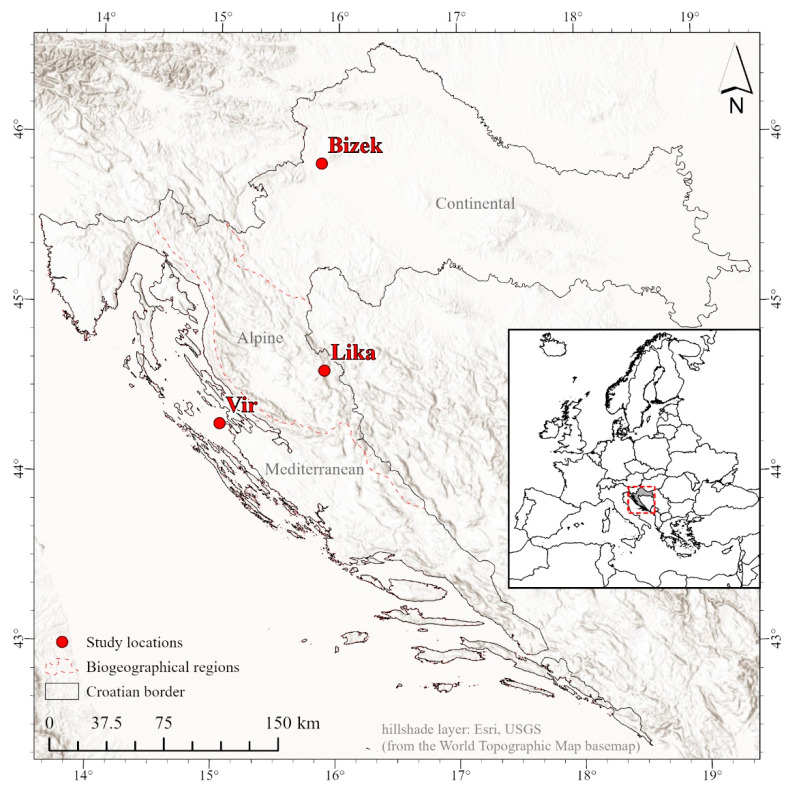
Map of Croatia with the locations where the research took place. Created in ArcGIS Pro (v. 3.5.4, Esri). The biogeographical regions layer according to the EEA [43]. Inset: position of Croatia in the Euro-Mediterranean area, with the extent of the main map indicated with the red dashed square.

The study was conducted at three sites. All coordinates are in WGS 1984 (EPSG:4326); elevations are in meters above sea level (m a.s.l.): Bizek quarry on Medvednica Mt. (hereafter “Bizek”, N: 45.838997°, E: 15.858436° ≈ 250–370 m a.s.l.), Malo Kamensko in Lika (hereafter “Lika”, N: 44.619048°, E: 15.894599° ≈ 980–1050 m a.s.l.), and Vir Island (hereafter “Vir”, N: 44.301197°, E: 15.034497° ≈ 1–40 m a.s.l.). These sites were selected to span the geographic range of the NW clade in Croatia [40] and to represent distinct climatic and vegetation zones (Figure 2 and Figure 3).

The Bizek site is situated in the Continental biogeographical region, at the very edge of Croatia’s capital city, Zagreb, and is characterized by a temperate continental climate with pronounced seasonal variation. Following the cessation of mining, this abandoned quarry has undergone secondary succession by mixed deciduous trees (*Fagus sylvatica*, *Quercus pubescens*, *Fraxinus* sp., *Populus* sp., *Salix* sp., etc.), and thermophilic shrubs like *Cronus sanguinea*, *Rubus* sp., and *Crataegus* sp. It is the most closed of the three study sites. No livestock grazing or management is present.

The Lika site is situated in the Alpine biogeographical region and is characterized by cooler conditions associated with higher elevation and Alpine influence. It is a part of the Veliko and Malo Kamensko grasslands; therefore, it is the most open of the three sites. Small-to-medium-sized rock piles are localized, usually in combination with shrubbery, while more extensive rock formations are scarce. Perennial grazing by cattle and occasional mowing are both present (Ml. Zadravec, pers. obs. 2017–2021).

The Vir site is situated in the Mediterranean biogeographical region, on the southwestern side of Vir Island. The climate is characterized as moderately warm and humid, with a pronounced dry period during the warmest months [42]. The site itself is mostly a former grassland overgrown with maquis and garrigue, intersected by several dry-stone walls. The western and southwestern parts of the site are covered with a forest, consisting mostly of *Quercus ilex* and *Pinus* sp. More open spaces are still present in parts, but are becoming increasingly rare due to vegetation succession. In terms of habitat openness, it is between the two other sites. There is no livestock grazing nor mowing at the site.

### 2.3. Data Gathering

We followed the methods described by Zadravec [31] and Zadravec et al. [44]. Field surveys were conducted between 7:30 and 20:00 h, with additional surveys taking place until 23:00 h to assess nocturnal activity. All times are reported in Central European Summer Time (UTC + 2). Active or hidden snakes were captured by hand using welding gloves THT1 TER25010 (Delta Plus, Domus Sesvete d.o.o., Zagreb, Croatia) from May to September 2017–2021.

Within two minutes of capture, T_b_ of each viper was measured in shade by gently inserting the tip of a temperature probe 52226 Dual Temp Infrared Probe Thermometer (Mastercool, Con-testor, Zagreb, Croatia) into the cloacal opening. Each snake was then placed in an individually numbered cloth bag for subsequent data gathering. Concurrently, eight environmental parameters were measured and recorded: substrate temperature (TS), air temperature at 5 cm (TA5) above the snake’s position, air temperature at 60 cm (TA60), relative humidity, atmospheric pressure, cloud cover, wind speed, and microhabitat. Additional data recorded at capture included time of day, behavior, and GPS coordinates with elevation (Garmin eTrex 30x). Each capture location was marked with a uniquely numbered colored cloth ribbon. Between search intervals, snout–vent length (SVL) was measured, and sex was determined. Stomach contents were assessed by gentle palpation of the ventral surface to detect the presence or absence of palpable prey items (hereafter: food in stomach) [45,46,47]. Pre-ecdysis condition (opaque skin) was also recorded (see Table A1 in Appendix A for details). For reliable individual identification, the posterior margins of ventral scales were clipped [31,48]. As supplementary identification, standardized photographs (Canon PowerShot SX70HS, Canosa, Zagreb, Croatia) were obtained for each individual. Photographs documented the head (dorsal, ventral, anterior, and lateral views), dorsal pattern, and clipped scale markings.

### 2.4. Statistical Analysis

**Figure A1:**
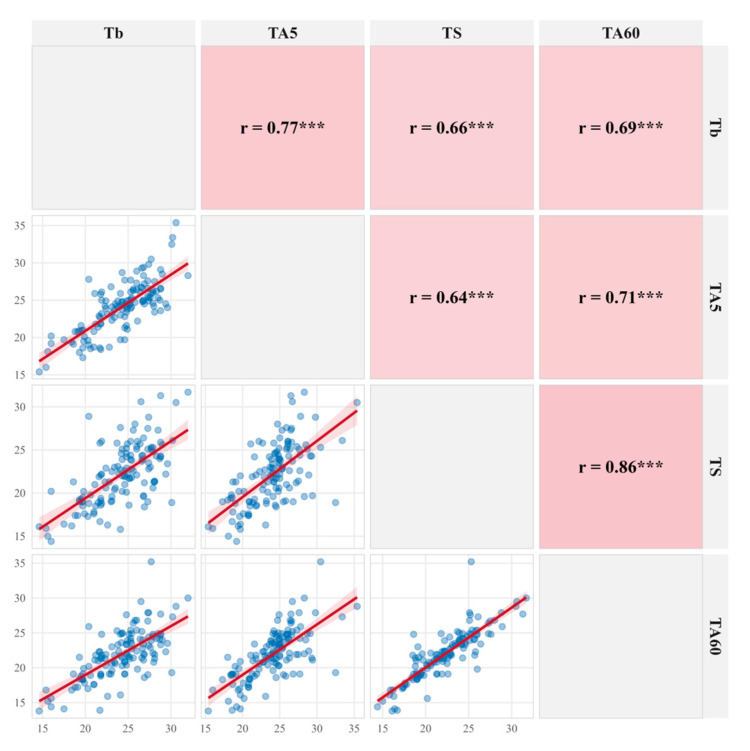
Pearson correlation matrix of body temperature (T_b_), air temperature at 5 cm (TA5), substrate temperature (TS), and air temperature at 60 cm (TA60). Color intensity represents the strength of the correlation, ranging from blue (negative) to red (positive). Correlation coefficients are displayed within each cell, with significance levels indicated by asterisks (*** *p* < 0.001). All pairwise correlations were statistically significant (*p* < 0.001).

Statistical analyses were conducted in RStudio (v. 2023.06.0, Posit software) using R (v.4.4.2) [49,50], with packages MuMIn [51], dplyr [52], and car [53]. All plots were generated using the ggplot2 package [54]. Individuals that had not yet undergone their first hibernation were excluded from all analyses. *Vipera ammodytes* exhibits a *berus*-type spermatogenesis cycle, with mating restricted to spring and parturition occurring the subsequent autumn. Consequently, any snakes encountered in early spring were considered post-hibernation and included in the analyses. We excluded gravid animals from datasets, since only seven gravid females were caught during the study (two at Bizek, five at Lika, none at Vir). Substrate (TS), air temperature at 5 cm (TA5), and at 60 cm (TA60) were strongly correlated (Figure A1). Performed variance inflation factors with TA60 reached 5.62, which exceeds standard thresholds; therefore, we excluded TA60. Preliminary full model analysis with both TS and TA5 gives the same model output as the model presented in this manuscript, but the preliminary full model with TS included pushes the n/k ratio down to 3.1, which is well below what’s recommended for stable parameter estimation, and the added complexity buys us no new insight. Since TA5 had the larger effect in preliminary full model analysis (4.82 °C per 2SD vs 1.68 °C per 2SD for TS), we retained only TA5 as it captures the dominant thermal signal. Following data screening, we standardized continuous predictors and identified influential observations to ensure model stability. Prior to modeling, we calculated descriptive statistics (mean ± SD, range) for T_b_ and continuous predictors, stratified by categorical variables (location, sex, opaque, food in stomach). Descriptive summaries verified data quality and aided interpretation of model-averaged estimates, which are expressed relative to reference levels for categorical variables.

Prior to analysis, we identified influential observations using Cook’s distance with a threshold of 4/n, removing seven observations that disproportionately affected model estimates: four from Vir, two from Bizek, and one from Lika. Continuous variables were standardized by centering on the mean and dividing by two standard deviations, following Gelman [55]. This standardization places regression coefficients for continuous predictors on a comparable scale with coefficients for binary predictors, facilitating interpretation of relative effect sizes. Categorical variables (location, microhabitat, sex, behavior, opaque, and food in stomach) were retained on their original scales.

We used an information-theoretic approach with model averaging [56,57] to identify variables influencing T_b_. This approach was chosen because multiple biologically plausible models exist for explaining thermoregulatory variation, and model averaging provides parameter estimates that account for model selection uncertainty.

The global linear model includes all measured biologically relevant predictors as main effects: location, air temperature at 5 cm above the snake’s location (TA5), relative humidity, wind speed, microhabitat type, time of day, snout–vent length (SVL), sex, behavioral state, pre-ecdysis condition (opaque), and presence of food in the stomach. We also included two-way interactions between location and all other predictors to test for location-specific effects.

All possible submodels were generated from this global model using the dredge function in the MuMIn package [51]. Models were ranked using the Akaike Information Criterion corrected for small sample size (AICc). We defined the top model set as all models with ΔAICc < 2, as models within this range have substantial empirical support. Relative variable importance was calculated as the sum of Akaike weights across all models in the top set containing each variable, providing a measure of how consistently each predictor improved model fit [56].

Model adequacy was assessed using residuals calculated from model-averaged predictions. We evaluated normality of residuals using Shapiro–Wilk tests and visual inspection of Q-Q plots, recognizing that formal tests are overly sensitive with moderate sample sizes. Homoscedasticity was assessed by examining residuals versus fitted values plots and testing the correlation between absolute residuals and fitted values. Multicollinearity among predictors was evaluated using variance inflation factors (VIF) calculated from a main-effects-only model to avoid artificial inflation from interaction terms; all VIF values were below 5, indicating no problematic collinearity. Model fit was quantified using the coefficient of determination (R^2^) calculated from model-averaged predictions and root mean square error (RMSE).

Parameter estimates were obtained by averaging across the top model set using the natural averaging method, whereby parameters are averaged only over models in which they appear [56,57]. Natural averaging was preferred because our primary interest was in estimating effect sizes conditional on predictor inclusion. Unconditional standard errors were calculated to incorporate both sampling variance and model selection uncertainty.

We assessed the robustness of our model-averaged inferences using sensitivity analyses with three model selection criteria: (1) models within ΔAICc < 2, (2) ΔAICc < 4, and (3) cumulative Akaike weight ≥ 95%. Model-averaged parameter estimates, relative variable importance, and predictions were compared across these three sets to verify that inferences were not sensitive to the choice of selection threshold. Based on ΔAICc < 2, we operationally classified predictors with relative importance 0.75–1.00 as highly important, 0.50–0.74 as moderately important, and 0.25–0.49 as weakly important. These categories are intended as interpretive aids rather than formal statistical thresholds; the limitations of interpreting summed Akaike weights as measures of variable importance have been discussed elsewhere [58]. Predictors with a relative importance score of less than 0.25 were discarded from further analyses. For methodology on model diagnostics, see Appendix B and Figure A3, Figure A4 and Figure A5.

To facilitate interpretation of interaction terms, we calculated post hoc contrasts using model-averaged estimates. Within-location contrasts compared variable levels (e.g., microhabitat types) at the same location, while between-location contrasts compared locations within the same variable level. For continuous predictors involved in interactions with location, we calculated location-specific slopes and pairwise differences between locations. Standard errors for all contrasts were calculated using the delta method with the full variance–covariance matrix from the averaged model, properly accounting for parameter covariance. Statistical support for effects was assessed by examining whether 95% confidence intervals (estimate ± 1.96 × unconditional SE) excluded zero. We emphasize effect estimation and evidence weights rather than null hypothesis significance testing, though we note when 95% CIs exclude zero as indicating non-zero effects reliably.

Relative variable importance and confidence intervals address different questions. Relative variable importance reflects how consistently a variable improves model fit across the candidate set, while the confidence interval reflects the precision of the estimated effect. A variable may be consistently retained in top models yet have a wide confidence interval when sample sizes for particular categories are small.

We assessed population-dependent effects in two steps. First, we used relative variable importance to evaluate whether each interaction term was consistently retained in well-supported models. Second, for interactions with adequate support, we used model-averaged pairwise contrasts to describe the direction and magnitude of location-specific effects. This two-step approach is necessary because the information-theoretic framework does not provide a single omnibus test for complex categorical interactions. For guidance on interpreting standardized effect sizes, interaction terms, and relative variable importance, see Appendix C.1.

In addition, we used ANOVA (aov function, stats package [50]), to test for differences in T_b_ among locations and between sexes, including their interaction.

## 3. Results

We captured 135 unique *V. ammodytes* individuals over five years. After excluding records with missing data, gravid females, and recaptures, the final dataset comprised 128 individuals.

**Figure 4:**
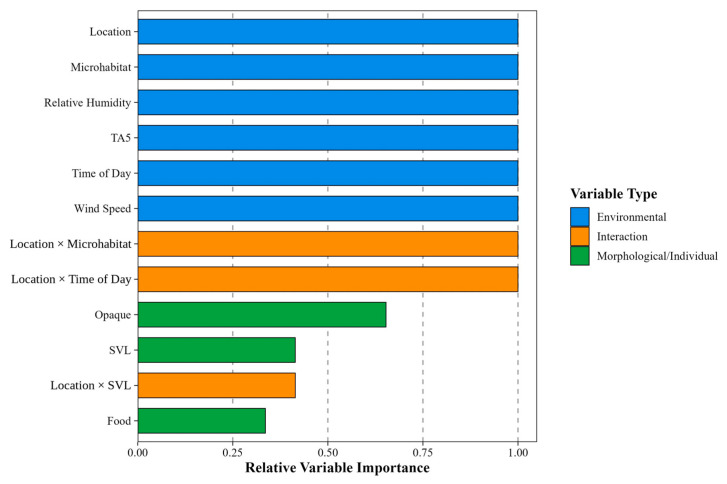
Relative importance of predictors influencing body temperature (T_b_) of *Vipera ammodytes*, including interactions, for models within ΔAICc < 2. TA5—air temperature at 5 cm above capture location; SVL—snout-to-vent length; Food—presence of food in stomach. Relative variable importance thresholds: 0.75–1.00: highly important, 0.50–0.74: moderately important, 0.25–0.49: weakly important.

**Figure A2:**
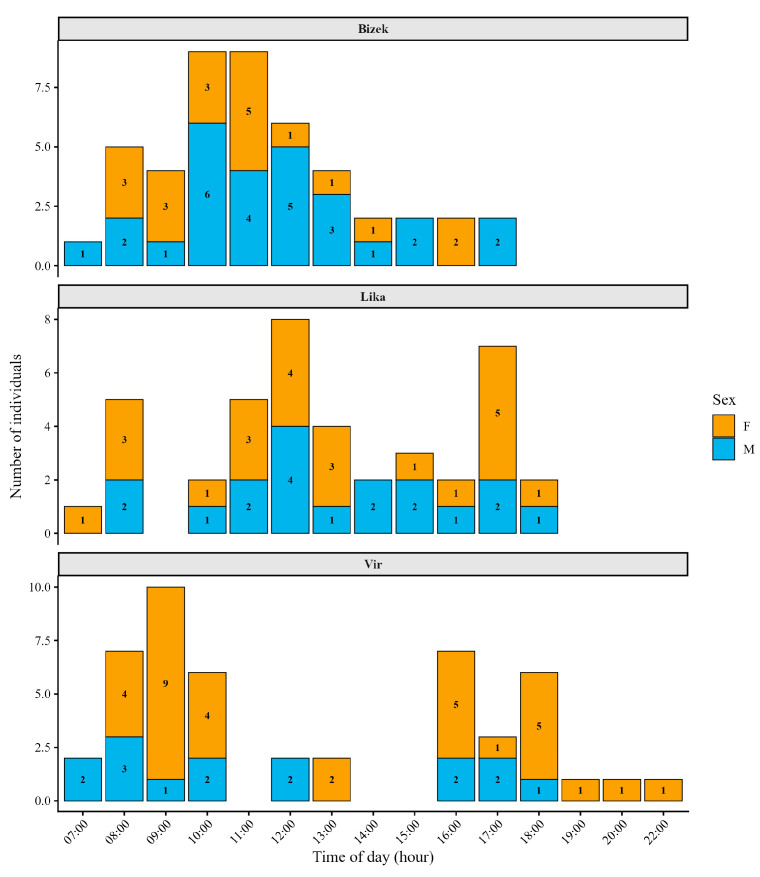
Number of individuals per sex and location found during each time slot, for all five years together.

**Figure A3:**
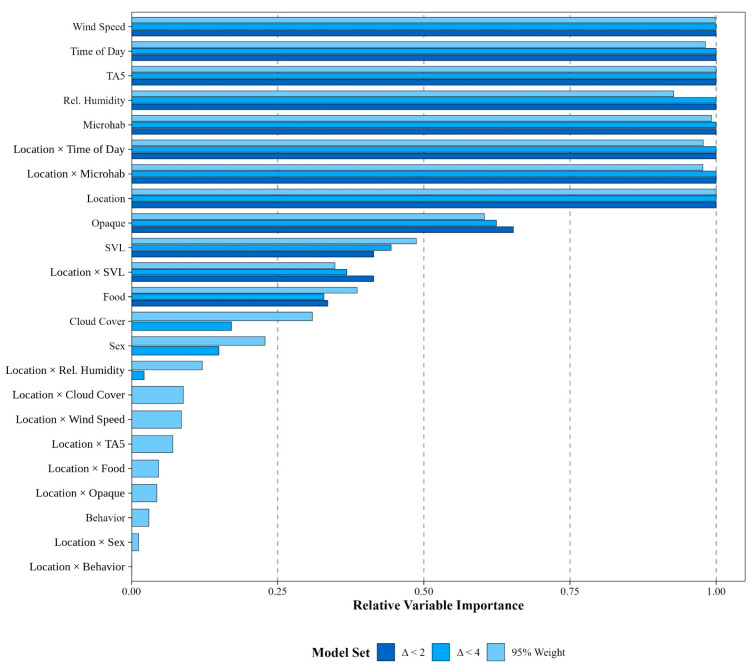
Importance of variables, including interactions, in the three model sets (AICc < 2, AICc < 4, and 95% weight) for all variables investigated for their influence on body temperature (T_b_) of *Vipera ammodytes*. TA5—air temperature at 5 cm above the place where the snake was found; SVL—snout to vent length; Food—presence of food in stomach.

**Figure A4:**
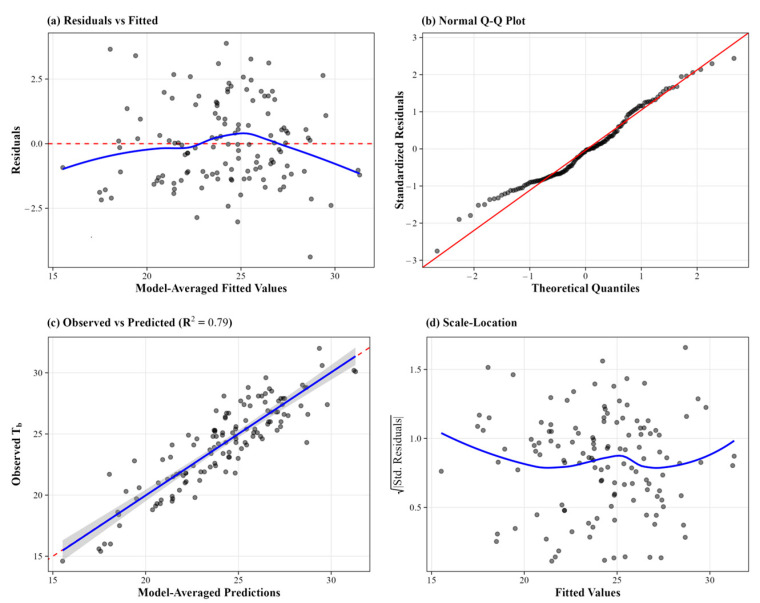
Model diagnostics (model-averaged predictions) for the AICc < 2 model set: (**a**) residuals vs. fitted, (**b**) normal Q-Q plot, (**c**) observed vs. predicted field body temperature (T_b_) values, (**d**) scale vs. location.

**Figure A5:**
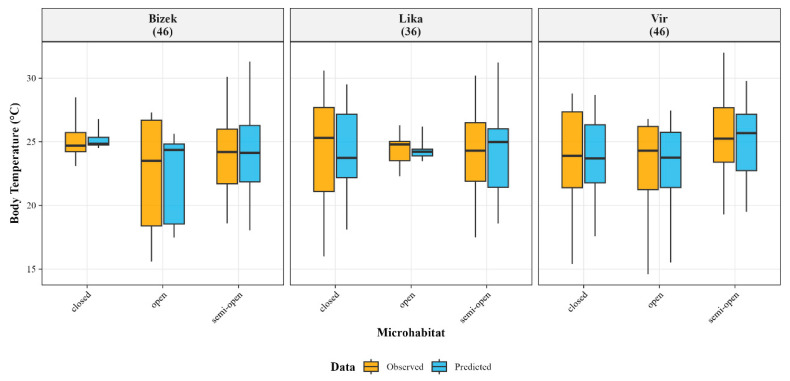
Raw field body temperature (T_b_) data vs. model-predicted T_b_ values.

Of the 12 investigated variables, model averaging identified nine supported predictors of T_b_, including three location interactions (Figure 4). Relative humidity, TA5, wind speed, opaque condition, and food in the stomach acted independently of location, whereas microhabitat, time of day, and SVL showed location-dependent effects. Relative humidity, TA5, and wind speed were highly important (relative variable importance = 1 for all three predictors), while opaque condition was moderately important (0.65), and food in stomach was weakly important (0.34). Among interaction terms, location × microhabitat and location × time of day were highly important (relative variable importance = 1 for both interactions), indicating that these location-dependent effects were consistently present in the top model set. Location × SVL received weaker importance (0.41). These values, reported also in Figure A3, are included here to clarify the model-level evidence before presenting pairwise contrasts. For descriptive datasets, see Figure A2, and Table A2 and Table A3.

**Figure 5:**
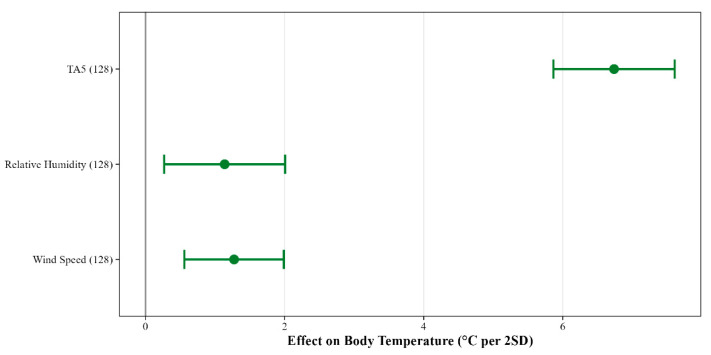
Model-averaged effects of air temperature at 5 cm (TA5), relative humidity, and wind speed on field body temperature (T_b_) in *Vipera ammodytes*. Effect sizes represent the change in field body temperature (°C) associated with a two-standard-deviation increase in each predictor variable. This standardization permits direct comparison of effect magnitudes across predictors measured on different scales. Total sample size is indicated in parentheses following each variable name. Points represent model-averaged estimates from the top model set (ΔAICc < 2), with error bars indicating 95% confidence intervals calculated using unconditional standard errors. Green points and error bars denote effects where the 95% CI excludes zero, indicating a statistically supported effect.

**Figure A6:**
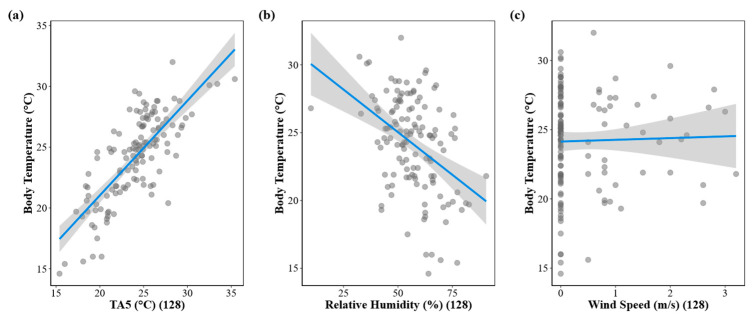
Relationships between continuous environmental variables and field body temperature (T_b_) in *Vipera ammodytes*. (**a**) Field T_b_ as a function of ambient temperature measured at 5 cm above substrate (TA5). (**b**) Field T_b_ as a function of relative humidity. (**c**) Field T_b_ as a function of wind speed. In all panels, points represent individual observations and lines indicate ordinary least squares regression fits with 95% confidence bands (shaded areas). Sample sizes are indicated in brackets on each x-axis label. The direction and magnitude of these relationships (positive vs. negative slopes) illustrate the raw correlational patterns in the data.

Individuals found at higher relative humidity had lower T_b_. In contrast, higher TA5 was associated with higher T_b_, while wind speed showed a slight positive association with T_b_ (Figure A6). Although all three environmental predictors were retained with high consistency across the top model set, their effect sizes differed considerably. TA5 had the largest effect (≈6.5 °C per 2SD), while relative humidity and wind speed (≈1 °C per 2SD) had much smaller effects (Figure 5). A high variable importance means the variable consistently contributes to model fit; it does not necessarily mean the variable has a large biological effect.

**Figure 6:**
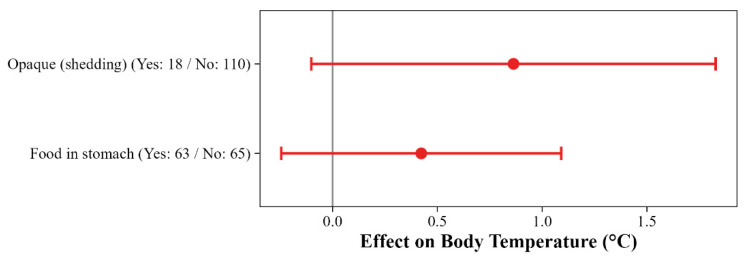
Model-averaged effects of pre-ecdysis condition (opaque) and recent feeding (food in stomach) on field body temperature (T_b_) in *Vipera ammodytes*. Effect sizes represent the difference in body temperature (°C) between individuals with and without each condition (i.e., the effect of ‘Yes’ relative to ‘No’). Sample sizes for each category are indicated in parentheses. Points represent model-averaged estimates from the top model set (ΔAICc < 2), with error bars indicating 95% confidence intervals. Red points and error bars denote effects where the 95% CI includes zero and indicate effects not statistically supported. Positive values indicate that individuals with the condition (opaque or food in the stomach) exhibited higher body temperatures than those without.

**Figure A7:**
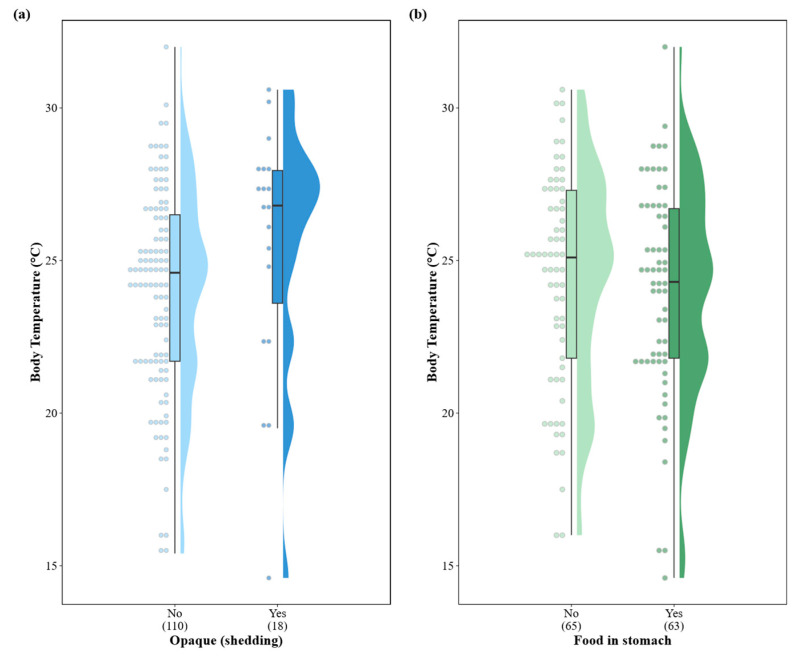
Field body temperature (T_b_) in relation to individual physiological state in *Vipera ammodytes*. (**a**) Field T_b_ distribution in individuals with and without signs of pre-ecdysis (opaque eyes and dull skin indicating imminent shedding). (**b**) Field T_b_ distribution in individuals with and without food in the stomach. Raincloud plots combine a half-violin showing the probability density (right), a box plot showing median (horizontal line), interquartile range (box), and min–max (whiskers), and individual observations as dots (left). Sample sizes for each category are indicated below the x-axis labels.

The influence of physiological states (the opaque condition and presence of food in the stomach) on T_b_ did not vary among locations. Although the opaque condition was moderately important and the presence of food in the stomach was weakly important across all model sets (Figure A3), 95% CIs included zero, indicating an insubstantial biological effect for both conditions. (Figure 6 and Figure A7).

**Figure 7:**
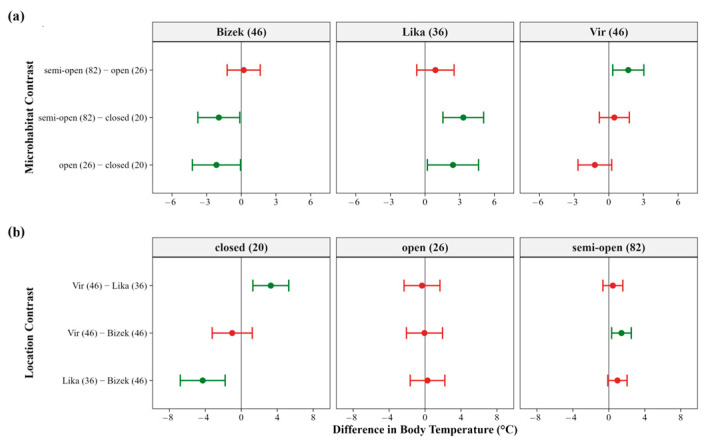
Model-averaged contrasts for field body temperature (T_b_) across locations and microhabitat types in *Vipera ammodytes*: (**a**) within-location microhabitat contrasts showing pairwise differences in T_b_ between microhabitat types within each study location. Each facet represents a single location, with sample size indicated in parentheses; (**b**) between-location contrasts showing pairwise differences in T_b_ among sites within each microhabitat type. Each facet represents a single microhabitat category, with sample size indicated in parentheses. In (**a**,**b**), contrast labels indicate the comparison (e.g., ‘Vir (n) − Bizek (n)’), where positive values indicate higher T_b_ at the first location/microhabitat type. Points represent model-averaged estimates from the top model set (ΔAICc < 2), and error bars indicate 95% confidence intervals calculated using unconditional standard errors. Green points and error bars indicate contrasts where the 95% CI excludes zero (statistically supported differences); red points and error bars indicate contrasts where the 95% CI includes zero (statistically unsupported differences).

**Figure A8:**
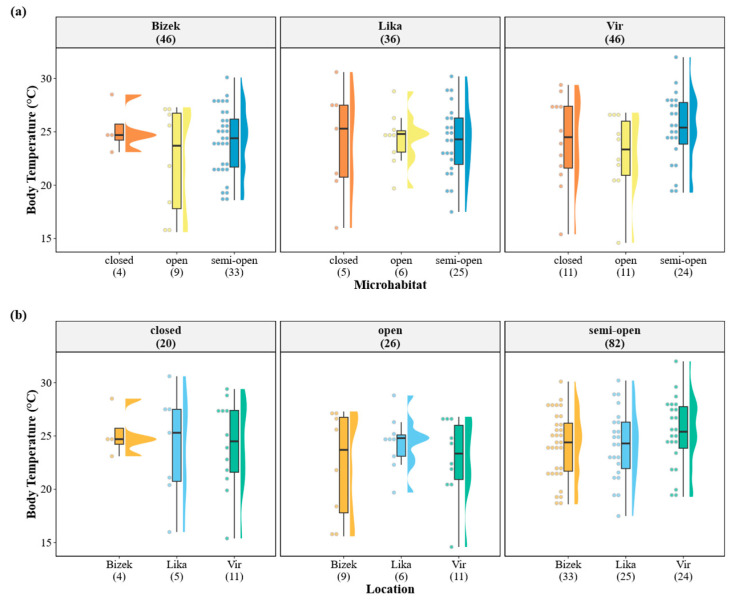
Body temperature variation across locations and microhabitat types in *Vipera ammodytes*. (**a**) Distribution of field body temperature (T_b_) by microhabitat type within each study location. (**b**) Distribution of field T_b_ by location within each microhabitat type. Raincloud plots combine a half-violin showing the probability density (right), a box plot showing median (horizontal line), interquartile range (box), and min–max (whiskers), and individual observations as dots (left). Each facet represents a single microhabitat category, with total sample size indicated in parentheses. Visualization elements as described for panel (**a**).

The influence of microhabitat on T_b_ varied among locations and was highly important across all models (Figure A3). At Bizek, T_b_ in closed microhabitats exceeded that in semi-open and open microhabitats. In contrast, at Lika, T_b_ in closed microhabitats was lower than in semi-open and open microhabitats. At Vir, T_b_ in semi-open microhabitats exceeded that in open microhabitats (Figure 7a and Figure A8a). Between-location comparisons indicated that T_b_ within the same microhabitat type varied among sites. Within closed microhabitats, T_b_ at Lika was lower than at Bizek and Vir. In semi-open microhabitats, T_b_ at Vir exceeded that at Bizek (Figure 7b and Figure A8b).

**Figure 8:**
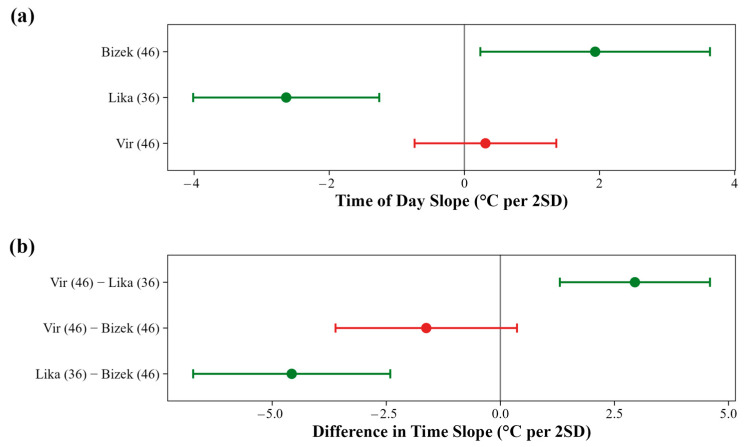
Location-specific effects of time of day on field body temperature (T_b_) in *Vipera ammodytes*: (**a**) model-averaged slopes describing the relationship between time of day and T_b_ at each site. Positive slopes indicate that the field body temperature increases throughout the day; negative slopes indicate decreasing T_b_. Slope magnitudes are expressed as a change in T_b_ (°C) per two standard deviations of time of day. Sample sizes for each location are indicated in parentheses. (**b**) Pairwise differences in time-of-day slopes between locations. Positive values indicate that the first location in each comparison exhibits a steeper (more positive) temporal slope than the second location. Contrast labels include sample sizes for each location. Points represent model-averaged estimates from the top model set (ΔAICc < 2), with error bars indicating 95% confidence intervals. Green points and error bars denote statistically supported effects (95% CI excludes zero); red points and error bars indicate effects not statistically supported.

**Figure A9:**
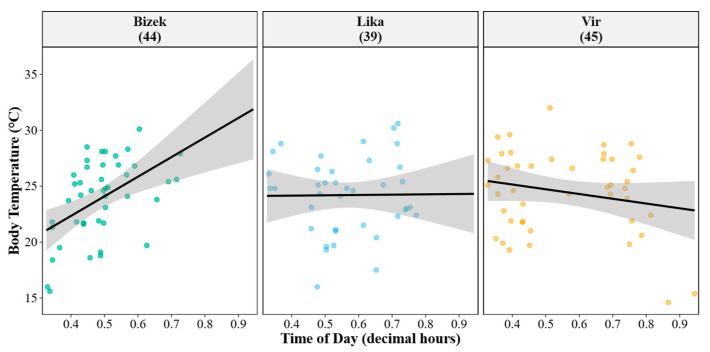
Relationship between time of day and field body temperature (T_b_) across locations in *Vipera ammodytes*. Each facet displays data from a single location (sample size in parentheses). Positive slopes indicate increasing field body temperature throughout the day; negative slopes indicate the opposite pattern. Points represent individual observations; lines indicate ordinary least squares regression fits with 95% confidence bands (shaded areas), extended across the full observed time range for visual comparison. Time of day is shown as decimal time.

Time of day was a highly important predictor, with location-dependent effects (Figure A3). At Bizek, T_b_ increased throughout the day, whereas at Lika, T_b_ decreased (Figure 8a and Figure A9). The temporal slope at Lika was lower than that at Bizek and Vir (Figure 8b).

**Figure 9:**
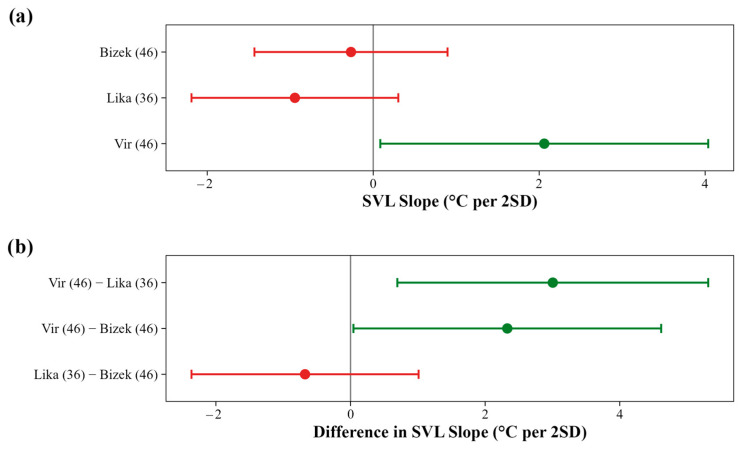
Location-specific effects of body size (SVL) on field body temperature (T_b_) in *Vipera ammodytes*. (**a**) Model-averaged slopes describing the relationship between snout–vent length and T_b_ at each study location. Positive slopes indicate that larger individuals exhibit higher T_b_; negative slopes indicate the opposite pattern. Slope magnitudes are expressed as a change in T_b_ (°C) per two standard deviations of SVL. Sample sizes for each location are indicated in parentheses. (**b**) Pairwise differences in SVL slopes between locations, testing the population × SVL interaction. Positive values indicate that the body size effect is stronger (more positive) at the first location relative to the second. Contrast labels include sample sizes for each location. Points represent model-averaged estimates from the top model set (ΔAICc < 2), with error bars indicating 95% confidence intervals. Green points denote statistically supported effects (95% CI excludes zero); red points indicate effects not statistically supported.

**Figure A10:**
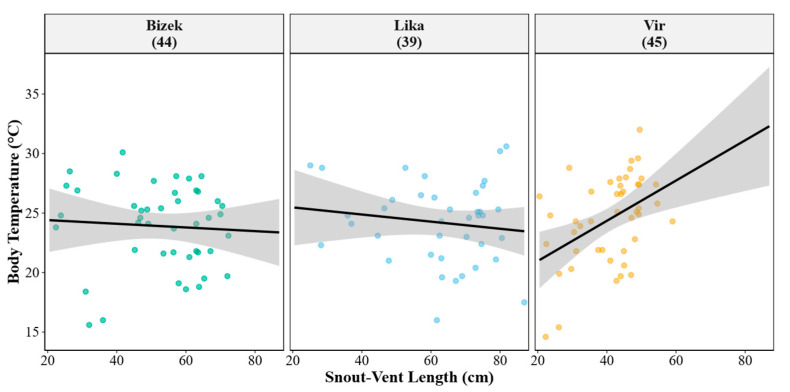
Relationship between body size (SVL) and field body temperature (T_b_) across locations in *Vipera ammodytes*. Each facet displays data from a single location (sample size in parentheses). Points represent individual observations; lines indicate ordinary least squares regression fits with 95% confidence bands (shaded areas). Positive slopes indicate that larger individuals exhibit higher field T_b_; negative slopes indicate the opposite pattern.

The effect of SVL on T_b_ varied among locations and was weak across all model sets (Figure A3). At Vir, larger individuals (greater SVL) exhibited higher T_b_, but the effect was relatively weak (≈2 °C per 2SD) (Figure 9a and Figure A10). The SVL–T_b_ relationship differed among locations. Specifically, the positive SVL–T_b_ slope at Vir was steeper than at Bizek or Lika (Figure 9b and Figure A10).

Cloud cover, sex, and behavior did not emerge as important predictors (Figure A3).

**Figure 10:**
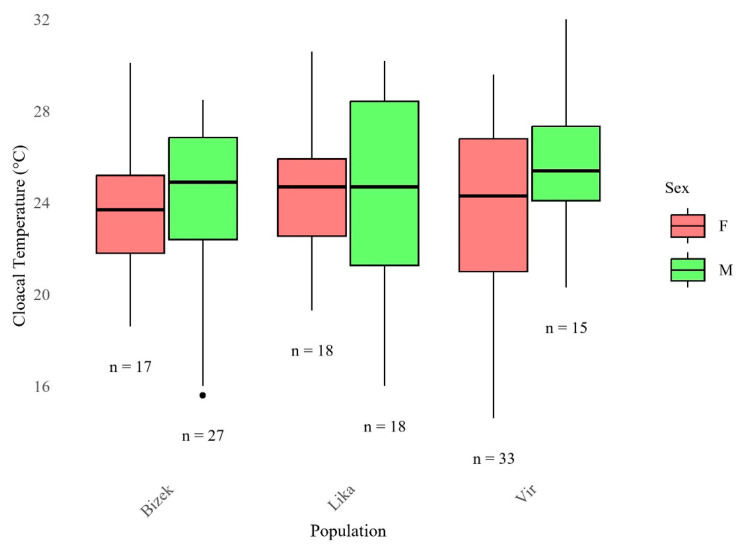
Distribution of field body temperature (T_b_) by sex and population. Sample sizes (n) shown beneath each boxplot. Boxes show medians and interquartile ranges; whiskers extend to minimum and maximum values. No statistical difference was found between groups (ANOVA).

Mean field T_b_ was similar across sites (range: 23.4–25.4 °C). The lowest recorded T_b_ was 14.3 °C for a male from Lika, while the highest was 32.0 °C for a male from Vir (Figure 10 and Table A4).

## 4. Discussion

In our study, nine of 12 variables were identified as predictors of T_b_ in *V. ammodytes*, including three with location-dependent effects. While abiotic and physiological constraints acted as predictors across populations, cloud cover and sex were not supported as important ones. Microhabitat use and activity timing functioned as population-specific buffers, as did SVL, but with a weak effect, whereas discrete behavioral categories did not. These results partially support our hypothesis. Ultimately, the hypothesis that T_b_ is conserved across populations and sexes was supported.

### 4.1. Abiotic Population-Independent Predictors

Body temperature closely tracks ambient temperature in reptiles, particularly species relying on basking and surface activity [3,6]. Indeed, TA5 was the most consistent predictor of T_b_ in our study, with individuals encountered at higher TA5 exhibiting higher T_b_. This consistency of the TA5 effect across locations suggests that, despite substantial differences in vegetation structure and climate, *V. ammodytes* relies on broadly similar thermal cues to regulate T_b_. A close relationship between T_b_ and environmental temperatures has been reported in other snake species [59,60]. However, single environmental variables often explain only part of the variation in T_b_, reflecting the combined influence of multiple environmental and behavioral factors. In this context, it is important to note the significant correlation of TA5 with other temperatures measured in this study (TS and TA60), indicating possible high predictivity for other ambient temperatures as well. This reinforces the idea that ambient temperature acts as a universal constraint on thermoregulation, within which behavioral and habitat-mediated adjustments operate.

Relative humidity exerted a negative effect on T_b_ across all populations, with individuals in more humid conditions exhibiting lower T_b_. Although humidity is often considered a secondary variable in reptile thermoregulation, it can substantially influence heat exchange by modulating evaporative cooling, boundary layer properties, and the thermal characteristics of substrates and vegetation [2,21]. In snakes, higher humidity is frequently associated with shaded or sheltered microhabitats that offer reduced solar radiation and lower operative temperatures [61] which may explain the negative effect humidity has on T_b_ observed in this study.

Wind speed enhances convective heat loss in ectotherms, with most studies testing this effect on lizards [59,60,62]. Empirical studies explicitly addressing wind effects on snake body temperature are scarce. Wind speed is commonly recorded as a contextual variable in viper field studies [31,63,64,65], but elapid studies report weak or unsupported relationships between wind and activity or detectability [66,67]. Nevertheless, practical field experience and monitoring guidelines commonly recommend avoiding surveys during strong wind conditions, reflecting a widespread perception that wind alters snake behavior or detectability [68]. Unexpectedly, we found not a negative, but a positive association between wind speed and body temperature across all locations, but its biological effect was slight. Interestingly, although extremely rare, some studies reported similar positive effects of wind on T_b_ or basking behavior, and have emphasized the need for further research to elucidate this phenomenon [69,70]. This counterintuitive pattern likely reflects complex interactions between heat exchange, environmental heterogeneity, and behavior rather than a direct thermal effect [3,6,59,71]. Our findings demonstrate that, although slightly significant, wind speed is not thermally neutral for *V. ammodytes*, but this effect needs to be studied further before suggesting any mechanistic interpretation. Incorporating wind into thermoregulation models may be particularly important as climate change alters wind regimes alongside temperature and humidity [72].

### 4.2. Physiological Population-Independent Predictors

Elevated body temperatures during pre-ecdysis and digestion have been reported in several reptile species. During the opaque phase, this is commonly interpreted as facilitating skin renewal through increased metabolic and enzymatic activity [8,21]. In snakes, the opaque phase is typically associated with reduced mobility, impaired vision, and increased vulnerability to predation, potentially constraining behavioral thermoregulation and limiting access to preferred thermal microhabitats [21]. Postprandial thermophily is also well documented in snakes and other reptiles, where elevated body temperatures enhance digestive efficiency by accelerating gastric emptying, enzymatic activity, and nutrient assimilation [17,73,74]. Experimental studies on European vipers have shown that digestion proceeds more rapidly at higher temperatures, highlighting the physiological benefits of maintaining elevated T_b_ following feeding. However, expression of postprandial thermophily in free-ranging snakes is often constrained by ecological factors, including predation risk, habitat structure, and limited access to suitable thermal microhabitats [21]. Our results showed a moderate association rather than a dominant thermoregulatory predictor for both conditions, yet it also showed uncertainty of their biological effects, meaning that our results do not clarify if shedding and food in the stomach are connected to higher or lower T_b_. The weak and unsupported effect observed in this study suggests that although digestive state and opaque conditions may influence T_b_ opportunistically but do not dominate thermoregulation in *V. ammodytes* under field conditions.

### 4.3. Population-Dependent Predictors

Adjustments in microhabitat use and activity types (which acted as proxies of behavior) are reported as determinants of T_b_ in *V. ammodytes* in previous studies [20]. Microhabitat also emerged as an important population-dependent predictor in our study. Closed microhabitats were associated with higher T_b_ at Bizek but lower T_b_ at Lika, whereas semi-open microhabitats were associated with higher T_b_ at Vir. These contrasting patterns indicate that microhabitat categories must be interpreted relative to local vegetation structure, solar exposure, and thermal buffering capacity. Yet the differences among sites might be a reflection of uneven sample sizes. Indeed, some microhabitat categories (e.g., open and closed in Lika and Bizek) are represented with only a handful of records. This might indicate the rarity of those microhabitat categories at those sites, reflecting differences in habitat heterogeneity across locations. Another explanation could be that this is a reflection of the microhabitat preferences of the snakes, regardless of what is available at each location. A more detailed ecological study, which includes microhabitat and habitat preference analysis through the daytime cycle, would be useful to untangle microhabitat and T_b_ interaction in *V. ammodytes*. Nevertheless, those uncertainties do not diminish the significance of microhabitat as a predictor of T_b_, since the microhabitat × location interaction appears across all models in our analysis. Similar context-dependent microhabitat effects on T_b_ are reported in other vipers and reptiles in general, where identical habitat categories function as heat sources or thermal refuges depending on the climate and vegetation density [3,6,20,61,75]. Our findings extend this concept by demonstrating that such effects persist even within a single clade and a relatively limited geographic range.

Time of day emerged as another important population-dependent predictor of T_b_. At Bizek, T_b_ increased throughout the day, consistent with gradual warming in a forested environment where solar penetration is delayed. At Lika, T_b_ declined later in the day, likely reflecting the decrease in ambient temperatures that commonly occurs in mountain environments towards the evening. No statistically supported temporal effect was detected at Vir, suggesting that coastal conditions may dampen diel thermal gradients or constrain behavioral options. Temporal shifts in daily activity are key thermoregulatory mechanisms in reptiles [3,6,21,76], and our results show these mechanisms are expressed differently depending on local habitat characteristics for *V. ammodytes* as well.

Although behavior is widely recognized as a key component of snake thermoregulation [8,21,23], it did not emerge as an independent predictor of T_b_ in our models. In ectotherms, behavior often manifests through where and when individuals position themselves in the environment, rather than through discrete activity categories alone. Microhabitat selection and activity timing often determine exposure to TA5, humidity, wind, and solar radiation, thus capturing behavioral thermoregulation more effectively than the categorical behavioral variable. Similar patterns have been reported in other reptile studies, where environmental or spatial predictors outperformed explicit behavioral variables despite behavior being the underlying mechanism [3,6,61]. Together, these results suggest that behavioral thermoregulation in *V. ammodytes* may be expressed through spatial and temporal habitat use rather than as discrete behavioral states detectable as independent predictors. However, this finding must be interpreted with caution. The absence of a detectable effect among broad behavioral classes (e.g., basking, hiding, moving, resting, other) does not imply that behavioral thermoregulation is restricted exclusively to habitat selection and temporal activity. Field classifications of snake behavior used here lack the resolution to capture fine-scale postural adjustments. Ambush-foraging vipers frequently employ subtle mechanisms to manipulate heat exchange while remaining stationary, such as dorsoventral flattening to maximize solar absorption, altering the tightness of coils to minimize convective heat loss, or making minor adjustments in bodily orientation relative to the sun. Because these micro-behaviors were not distinctly categorized, their thermoregulatory contribution remains unquantified. Thus, while macro-scale spatial and temporal positioning are clearly vital, they likely operate in tandem with these more cryptic postural behaviors.

Body size effects on thermoregulation have been reported in several reptile taxa [77], including vipers [13,22], but their expression often depends on the environmental and ecological context. In our study, the relationship between SVL and T_b_ varied among populations and was statistically supported only at Vir, where larger individuals attained higher T_b_. Although detectable, the effect was comparatively weak, reinforcing the view that body size acts as a secondary modifier of thermal state rather than a dominant predictor. Moreover, the restriction of this effect to a single population suggests that size-related influences on thermoregulation are context-dependent rather than universally expressed.

### 4.4. Similarity of Body Temperatures Across Populations and Sexes

Despite pronounced differences in climate, vegetation, and predictor effects, mean T_b_ was similar across populations, with no difference between sexes. These similarities in mean T_b_ may nevertheless arise through different thermoregulatory pathways, as suggested by the contrasting temporal and microhabitat-related patterns observed among sites. For example, T_b_ increased throughout the day at Bizek but declined at Lika, indicating that snakes in different environments may rely on distinct combinations of habitat use and daily activity to achieve comparable thermal outcomes. Comparable results have been reported in vipers and some other reptiles, where population-specific thermoregulatory strategies, such as behavioral shifts to different microhabitats, may produce similar body temperatures despite divergent environmental conditions [14,33,64,75,78]. Yet it remains unclear whether these similarities in T_b_ across different *V. ammodytes* populations reflect active thermoregulatory convergence or simply similar environmental conditions across sites. Therefore, this needs to be studied further. The absence of sex-specific differences in T_b_ further suggests that, except for gravidity (not tested in this paper), males and females operate within similar thermal constraints, as it is demonstrated for other reptile species as well [22,79,80].

### 4.5. Implications for Conservation

The presence of both population-independent and population-dependent predictors of T_b_ has potential implications for the conservation and management of *V. ammodytes*. Our results demonstrate that even within a single genetic clade, populations achieve similar thermal outcomes through different combinations of environmental and behavioral pathways. This pattern is observed in other reptiles as well, supporting the view that context-dependent thermoregulatory pathways may be a common feature across ectotherms [14,23,75].

Population-dependent predictors, specifically microhabitat use, time of day, and SVL, indicate that the relative importance of particular thermal pathways varies spatially. Habitat management targeting vegetation structure, openness, and rock availability, or managing human activity times, might therefore benefit from being informed by site-specific thermoregulatory data. Without such context, interventions intended to improve habitat quality could inadvertently reduce the availability of effective thermal refuges. Given contrasting land-use contexts among sites, uniform management actions may be less effective. Instead, managers should consider how local environmental and anthropogenic factors interact with diel activity patterns [81,82,83]. For instance, preserving a heterogeneous semi-open microhabitat structure would likely maintain current thermoregulatory opportunities across all three sites; however, achieving this may require different local approaches, such as preventing excessive succession at Bizek, adjusting the timing and intensity of livestock grazing at Lika, and maintaining the existing mosaic structure through selective clearing at Vir.

At the same time, environmental predictors consistent across populations (TA5, relative humidity, wind speed) highlight shared constraints operating throughout the species’ range. Yet these predictors are likely tied to habitat structure [3,76]. Microhabitat composition and heterogeneity strongly influence air temperatures, moisture retention, and airflow, thereby shaping the microclimatic conditions experienced by snakes at fine spatial scales. Heterogeneous habitats with open, semi-open, and closed structures provide thermal and hydric gradients. These gradients likely allow individuals to buffer against short-term environmental variation and select conditions meeting their targeted thermal ranges [21].

Ongoing global warming, increased frequency of heat extremes, and altered precipitation and wind regimes are expected to reduce the availability of suitable thermal conditions for many ectotherms [5,22,84,85]. Combined with findings from other clades [20], our results suggest that the capacity of *V. ammodytes* to adjust to regional climate trajectories may depend heavily on retention of already mentioned fine-scale habitat heterogeneity [86]. Therefore, habitats lacking structural complexity may limit options available to vipers to manage their heat exchange, potentially altering their activity windows and energy budgets even if average climatic conditions remain within tolerable limits.

### 4.6. Strengths, Limitations, and Future Directions

A major strength of this study lies in its multi-year, multi-population design and its use of an information-theoretic framework that accounts for model uncertainty. By concurrently evaluating the environmental, behavioral, physiological, and morphological factors, we comprehensively identified predictors of T_b_ in *V. ammodytes*. Given the centrality of thermoregulation to ectotherm fitness, our results highlight the importance of identifying specific T_b_ predictors as an important step for understanding reptile thermal physiology [22,23].

Nevertheless, some limitations warrant consideration. Sample sizes were uneven among time of day, microhabitats, and populations, potentially limiting inference for rare categories. Because our measurements lack key environmental drivers, such as solar radiation or operative temperature, this limits our ability to draw clear mechanistic conclusions about the observed relationships. Because the descriptive figures show unadjusted data while the model-averaged contrasts account for covariate imbalance and interactions, the two will not always align visually. We therefore present raw distributions to illustrate sampling structure, but base inference on model-averaged estimates that control for confounding predictors.

In future studies, direct measurements of operative environmental temperatures combined with microclimatic profiles and habitat preference, and long-term individual tracking would further refine the understanding of thermoregulatory decisions. Future studies should expand this approach across additional clades and regions, incorporate fine-scale thermal mapping, and explore fitness consequences of thermoregulatory strategies under projected climate scenarios [22,23]. A comprehensive assessment of habitat availability would strengthen inferences by enabling analysis of preferred vs. available habitat selection.

## 5. Conclusions

Our study demonstrates that TA5, wind speed, and relative humidity act as universal constraints on thermoregulation in *V. ammodytes*, whereas pre-ecdysis condition and the presence of food in the stomach exert weaker effects. In contrast, the importance of microhabitat use, time of day, and SVL varied among populations. Despite differing predictor effects, mean T_b_ was similar across populations, confirming that comparable thermal outcomes arise through population-specific ecological pathways.

These findings reveal a combination of conserved and population-specific regulatory mechanisms that enable *V. ammodytes* to occupy diverse environments. Such flexibility may facilitate persistence under ongoing environmental change. At the same time, this persistence depends on the availability of fine-scale habitat structures. From a broader perspective, this reinforces that thermal physiology in ectotherms must be interpreted in light of local adaptation and site-specific environmental conditions.

Conservation-wise, management strategies that account for population-specific thermoregulatory pathways are essential for the long-term persistence of *V. ammodytes* across its range, which may be applicable to other reptiles as well.

## Data Availability

The dataset and R code for this paper are available at https://doi.org/10.6084/m9.figshare.28787801.v1.

## Appendix A

### Appendix A.2. Supplementary Tables

**Table A1:** List and description of environmental, morphometric, physiological, and behavioral variables of snakes used in the analyses. *—parameters kept in further analyses after initial inspection for correlations and subsequent interpretation based on ΔAICc < 2.

Parameter	Unit of Measurement	Obtained By	Description
Body temperature (T_b_) *	°C	Mastercool 52226 Dual Temp Infrared Probe Thermometer	field body temperature of the snake measured within one minute of capture, by gently inserting the tip of the thermometer’s temperature probe into the cloaca just beneath the anal scale; if needed, that part of the body was artificially shaded from direct sunlight while the measurement took place
Substrate temperature (TS)	°C	Mastercool 52226 Dual Temp Infrared Probe Thermometer	substrate temperature measured at the position where each snake was found, using the tip of the thermometer’s temperature probe; if needed, the tip was artificially shaded from direct sunlight while the measurement took place
Air temperature at 5 cm (TA5) above the snake’s position *	°C	Kestrel 4500 NV pocket weather tracker (Kestrel Instruments, RowingShop, Zagreb, Croatia)	air temperature measured at 5 cm above the position where each snake was found, using the tip of the thermometer’s temperature probe; if needed, the measuring device was artificially shaded from direct sunlight and the wind while the measurement took place
Air temperature at 60 cm (TA60)	°C	Mastercool 52226 Dual Temp Infrared Probe Thermometer or Kestrel 4500 NV pocket weather tracker	air temperature measured at 60 cm above the position where each snake was found, using the tip of the thermometer’s temperature probe in cases where the Mastercool was used; if needed, the measuring device was artificially shaded from direct sunlight and the wind while the measurement took place
Relative humidity *	%	Kestrel 4500 NV pocket weather tracker	relative humidity measured at 5 cm above the position where each snake was found; if needed, the measuring device was artificially shaded from direct sunlight and the wind while the measurement took place
Cloud cover	%	visual estimation	percentage of sky that was obscured by clouds, as observed from the position where each snake was found, estimated by eye and rounded to the nearest ten percent
Wind speed *	m/s	Kestrel 4500 NV pocket weather tracker	wind speed measured at 80 cm above the position where each snake was found, measured immediately after capture
Microhabitat *	(none)	direct field observation	a categorical variable assigned to a 1 m × 1 m square centered around positions for each snake where it was found, it can be one of three states: open (little or no vegetation cover, meaning snakes were quite easy to observe), semi-open (a moderate amount of vegetation cover, the snakes were partially concealed and not so easily observed) or closed (dense vegetation providing substantial amount of cover, the snakes were almost or completely imperceptible)
Time of day *	hours and minutes	direct field measurement	a DateTime variable indicating when each individual was found, recorded by checking the time on mobile phones, to the precision level of 1 min.
Sex *	(none)	direct field observation	a categorical variable assigned to each individual, denoting the sex of each caught snake
Behavior	(none)	direct field observation	a categorical variable assigned to each individual, denoting the observed behavior at the exact time the individual was spotted. Basking—the snake was coiled up in direct sunlight; Hiding—the snake was concealed under an object, e.g., a rock or roof tile, and was observed only when the object was turned over; Moving—the snake was actively moving about; Resting—the snake was out in the open, but resting in the shade; Other—any other behavior not covered by the aforementioned categories
Food in stomach *	(none)	direct field observation	a categorical variable assigned to each caught individual. Y—food was detected by palpation of the stomach; N—food was not detected by palpation of the stomach. A lack of detection by palpation does not necessarily mean that the stomach is empty, but larger (relative to the snake’s size) or undigested prey items can be reliably detected by this method.
Opaque (pre-ecdysis) *	(none)	direct field observation	a categorical variable assigned to each caught individual. Y—the snake was opaque, also called “in blue”, i.e., in the process of preparing to shed its skin; N—the snake was not opaque
Snout to vent length (SVL) *	cm	direct field measurement	a numerical variable measured to a precision of 0.1 cm using a narrow flexible tape measure

**Table A2:** Frequency of food presence and opaque condition by location and sex. Values are count/total (%).

		F			M			Total	
Location	Total N	N	Food (+)	Opaque (+)	N	Food (+)	Opaque (+)	Food (+)	Opaque (+)
Bizek	46	19	12/19 (63.2%)	1/19 (5.3%)	27	11/27 (40.7%)	2/27 (7.4%)	23/46 (50%)	3/46 (6.5%)
Lika	41	23	6/23 (26.1%)	7/23 (30.4%)	18	7/18 (38.9%)	5/18 (27.8%)	13/41 (31.7%)	12/41 (29.3%)
Vir	48	33	20/33 (60.6%)	4/33 (12.1%)	15	10/15 (66.7%)	1/15 (6.7%)	30/48 (62.5%)	5/48 (10.4%)

Food (+) = food item present in the stomach; Opaque (+) = snake is in the opaque (pre-ecdysis) condition. Values are count/total (%).

**Table A3:** Body temperature (T_b_) and snout–vent length (SVL) by location, time of day, behavior, and sex.

				F			M		
Location	Time of Day	Behavior	Total N	N	Tb (°C)	SVL (mm)	N	Tb (°C)	SVL (mm)
Bizek	Morning (<10:00)	Basking	5	3	23.4 ± 2	54.9 ± 7.1	2	19.8 ± 5.3	35 ± 1.4
	Morning (<10:00)	Moving	4	3	22.4 ± 3.3	67.2 ± 2	1	18.4	31
	Morning (<10:00)	Resting	1	0	—	—	1	15.6	32
	Midday (10:00–14:00)	Basking	18	8	22.5 ± 3.1	57.3 ± 7.9	10	25.4 ± 2.7	54.2 ± 13.1
	Midday (10:00–14:00)	Moving	5	0	—	—	5	24.4 ± 2.6	46.5 ± 19
	Midday (10:00–14:00)	Other	2	2	26.2 ± 3	51.5 ± 16.3	0	—	—
	Midday (10:00–14:00)	Resting	3	0	—	—	3	25.7 ± 2.3	51.5 ± 23.9
	Afternoon (14:00–18:00)	Basking	2	1	22.6	72.5	1	23.8	22.4
	Afternoon (14:00–18:00)	Moving	3	0	—	—	3	24 ± 3.8	60.2 ± 13.8
	Afternoon (14:00–18:00)	Other	2	2	27.8 ± 3.3	47.2 ± 7.8	0	—	—
	Afternoon (14:00–18:00)	Resting	1	0	—	—	1	27.9	60.9
Lika	Morning (<10:00)	Basking	6	4	26 ± 1.6	54.1 ± 15.9	2	28.8 ± 0	53.4 ± 35.1
	Midday (10:00–14:00)	Basking	11	6	23.6 ± 2.6	67.4 ± 5.7	5	23.3 ± 2.9	60 ± 15.3
	Midday (10:00–14:00)	Moving	2	1	21	47.8	1	25.3	65.5
	Midday (10:00–14:00)	Other	3	3	25.1 ± 2.3	69.7 ± 6.6	0	—	—
	Midday (10:00–14:00)	Resting	3	1	21.1	78.8	2	17.9 ± 2.6	65.3 ± 5.2
	Afternoon (14:00–18:00)	Basking	10	7	24.9 ± 3.3	71.3 ± 11.7	3	29.3 ± 0.8	52.6 ± 27.5
	Afternoon (14:00–18:00)	Other	3	0	—	—	3	23.7 ± 3.1	54.4 ± 23.9
	Afternoon (14:00–18:00)	Resting	1	0	—	—	1	17.5	87
	Evening (≥18:00)	Basking	1	1	22.4	74.6	0	—	—
	Evening (≥18:00)	Moving	1	0	—	—	1	23.1	44.6
Vir	Morning (<10:00)	Basking	10	8	24.3 ± 3.1	49.2 ± 5.8	2	26.9 ± 3.6	53 ± 8.4
	Morning (<10:00)	Moving	7	5	25.4 ± 4	42.6 ± 9.4	2	22.7 ± 3.4	39.1 ± 13.4
	Morning (<10:00)	Resting	2	0	—	—	2	24.6 ± 3.8	41.3 ± 3.7
	Midday (10:00–14:00)	Basking	3	1	23.4	30.5	2	24.6 ± 4	49.5 ± 6.6
	Midday (10:00–14:00)	Moving	4	3	20.8 ± 1.1	38.7 ± 6.8	1	32	49.5
	Midday (10:00–14:00)	Other	1	0	—	—	1	26.8	35.5
	Midday (10:00–14:00)	Resting	2	2	25.5 ± 1.6	39.1 ± 5.3	0	—	—
	Afternoon (14:00–18:00)	Basking	2	2	26.1 ± 1.8	49.2 ± 0.1	0	—	—
	Afternoon (14:00–18:00)	Moving	1	0	—	—	1	25.1	43
	Afternoon (14:00–18:00)	Other	5	3	26.5 ± 2	41.2 ± 9.2	2	24.6 ± 1.1	40.8 ± 12
	Afternoon (14:00–18:00)	Resting	2	1	24.8	23.6	1	28.7	46.7
	Evening (≥18:00)	Basking	1	1	19.8	47	0	—	—
	Evening (≥18:00)	Moving	4	4	21.6 ± 5.3	30.8 ± 9.8	0	—	—
	Evening (≥18:00)	Resting	4	3	21.6 ± 6.8	33.4 ± 10.2	1	26.4	20.5

Values are mean ± SD. ‘—’ = no observations. Single observations shown without SD. Tb = body temperature; SVL = snout–vent length.

**Table A4:** Summary of body temperatures (T_b_) for males and females per population. n—sample size; SD—standard deviation.

Sex	Location	n	Minimum (°C)	Maximum (°C)	Mean (°C)	Median (°C)	SD
	Bizek	17	18.6	30.1	23.4	23.7	3.12
♀	Lika	18	19.3	30.6	24.3	24.7	2.92
	Vir	33	14.6	29.6	23.8	24.3	3.75
	Bizek	27	15.6	28.5	24.1	24.9	3.63
♂	Lika	18	16.0	30.2	24.2	24.7	4.30
	Vir	15	20.3	32.0	25.7	25.4	3.10

## Appendix B

### Appendix B.1. Supplementary Methods for Model Diagnostics

Model validation for the information-theoretic framework.

Standard diagnostic procedures for single-model inference require adaptation when applied to multi-model frameworks. Following recommendations for information-theoretic analyses, we assessed model adequacy using residuals derived from model-averaged predictions rather than from any single model [56].

#### Appendix B.1.1. Calculation of Model-Averaged Residuals

Model-averaged predictions were calculated as the weighted sum of predictions from all models in the top set (ΔAICc < 2), with weights proportional to each model’s Akaike weight renormalized to sum to unity. Residuals were then computed as the difference between observed T_b_ and these model-averaged predictions. This approach ensures that diagnostic assessments reflect the actual inferential framework employed rather than any single approximating model.

#### Appendix B.1.2. Assessment of Model Fit

Overall model fit was quantified using the coefficient of determination (*R*^2^) ± calculated from model-averaged predictions:

(A1) $$R^{2}=1-\frac{SS\_residual}{SS\_total}$$

where *SS_residual* is the sum of squared model-averaged residuals and *SS_total* is the total sum of squares. We also report root mean square error (RMSE) as a measure of predictive accuracy in the original units of measurement. For comparison, we present the range of *R*^2^ values across individual models in the top set and the weighted mean *R*^2^ using Akaike weights.

#### Appendix B.1.3. Residual Diagnostics

Normality of model-averaged residuals was assessed using the Shapiro–Wilk test and visual inspection of quantile–quantile (Q-Q) plots. We note that with moderate to large sample sizes, the Shapiro–Wilk test may detect minor deviations from normality that have a negligible impact on inference; therefore, visual assessment of Q-Q plots was prioritized over formal test results.

Homoscedasticity was evaluated by plotting residuals against fitted values and by calculating the Spearman rank correlation between absolute residuals and fitted values. A significant correlation would indicate heteroscedasticity, potentially warranting variance-stabilizing transformations or alternative error structures.

Residuals were also examined separately for each location to verify that model predictions did not exhibit systematic bias at particular study sites.

#### Appendix B.1.4. Multicollinearity Assessment

Variance inflation factors (VIF) were calculated to assess multicollinearity among predictors. Because VIF values for main effects are artificially inflated when interaction terms are present, we calculated VIF from a reduced model containing main effects only. For categorical predictors with more than two levels, generalized VIF values were computed and adjusted for degrees of freedom following Fox and Monette [87]. VIF values exceeding 5 were considered indicative of problematic multicollinearity requiring remediation; values exceeding 10 would indicate severe multicollinearity.

#### Appendix B.1.5. Influential Observations

Influential observations were identified using Cook’s distance calculated from the best-supported model (lowest AICc) as an approximation, given the computational burden of calculating influence measures across all models in the averaged set. Observations with Cook’s distance exceeding 4/n, where n is the sample size, were flagged as potentially influential. We report the percentage of observations exceeding this threshold and conducted sensitivity analyses excluding these observations to verify that conclusions were robust to their inclusion.

#### Appendix B.1.6. Diagnostic Figures

Four diagnostic plots were generated to visualize model adequacy (Figure A4): (Figure A4a) residuals versus fitted values, with a LOESS smoother to detect non-linearity; (Figure A4b) normal Q-Q plot of standardized residuals; (Figure A4c) observed versus predicted values with 1:1 reference line and R^2^ annotation; and (Figure A4d) scale–location plot showing the square root of absolute standardized residuals versus fitted values to assess homoscedasticity.

## Appendix C

### Appendix C.1. Interpretation of Model-Averaged Results

#### Appendix C.1.1. Understanding Effect Sizes from Standardized Predictors

Continuous predictor variables in this analysis were standardized by centering on their mean and dividing by two standard deviations prior to model fitting. Consequently, regression coefficients for continuous predictors represent the expected change in body temperature (°C) associated with an increase of two standard deviations in the predictor variable. This standardization approach, recommended by Gelman [55], offers two advantages: it places effect sizes for continuous predictors on a scale comparable to those for binary predictors (which inherently represent a one-unit change), and it facilitates comparison of effect magnitudes across predictors measured in different units.

To convert standardized effect sizes to the original measurement scale, divide the coefficient by two times the standard deviation of the predictor. For example, if ambient temperature has a standard deviation of 3.5 °C and the standardized coefficient is 6.0 °C per 2SD, the effect per 1 °C increase in ambient temperature is 6.0/(2 × 3.5) = 0.86 °C.

#### Appendix C.1.2. Interpretation of Interaction Terms

When a predictor is involved in an interaction with location, the main effect coefficient represents the effect of that predictor at the reference location (alphabetically first). The interaction coefficient represents the difference in slopes between a non-reference location and the reference location. To obtain the slope at a non-reference location, add the interaction coefficient to the main effect coefficient.

The within-location and between-location contrasts presented in the figures were calculated from these model-averaged coefficients to provide more directly interpretable comparisons. Standard errors for contrasts properly account for covariance between the main effect and interaction terms.

#### Appendix C.1.3. Relative Variable Importance

Variable importance values represent the sum of Akaike weights across all models in the top set that include the variable. Values range from 0 to 1, with higher values indicating that a variable consistently appeared in well-supported models. An importance value of 1.0 indicates the variable appeared in all models within the top set. Importance values should be interpreted as measures of predictor relevance rather than as probabilities or measures of statistical significance.

#### Appendix C.1.4. Statistical Support Versus Null Hypothesis Testing

We deliberately employ the language of ‘statistical support’ rather than ‘statistical significance’ to reflect the information-theoretic philosophy underlying this analysis. Effects are described as ‘supported’ when 95% confidence intervals exclude zero, indicating that the data provide evidence for a non-zero effect. Effects are described as ‘not supported’ when confidence intervals include zero, indicating insufficient evidence to conclude the effect differs from zero. This framing avoids the binary thinking associated with *p*-value thresholds while still providing clear guidance on which effects are reliably estimated [88].

### Appendix C.2. Supplementary Figures—Descriptive Statistics

Differences between raw and model-predicted variable values reflect covariate adjustment. The model-averaged estimates provide accurate comparisons by controlling for such imbalances.
